# Impact of vibrational strong coupling on liquid–liquid phase separation in supramolecular polymers

**DOI:** 10.1039/d5sc04149j

**Published:** 2025-08-07

**Authors:** Kripa Joseph, Hailin Fu, Joost J. B. van der Tol, Werner Steffen, Feixia Ruan, George Fytas, E. W. Meijer

**Affiliations:** a Institute for Complex Molecular Systems, Laboratory of Macromolecular and Organic Chemistry, Eindhoven University of Technology PO Box 513 Eindhoven 5600 MB The Netherlands e.w.meijer@tue.nl; b Department of Materials Science and Engineering, Department of Chemistry, Research Center for Industries of the Future, Westlake University 310030 Hangzhou China; c Max Planck Institute for Polymer Research Ackermannweg 10 55128 Mainz Germany fytas@mpip-mainz.mpg.de; d School of Material Science and Engineering, Zhejiang University 310030 Hangzhou China; e Faculty of Physics, Adam Mickiewicz University Uniwersytetu Poznanskiego 2 61-614 Poznan Poland; f Institute of Electronic Structure and Laser, FO.R.T.H Heraklion Greece

## Abstract

Liquid–liquid phase separation (LLPS) is a universal phenomenon that plays a key role in many biological processes. Although LLPS is well known for (bio)macromolecular systems, we have recently demonstrated that supramolecular polymer systems can also undergo LLPS *via* an entropy-driven pathway. This opens new avenues for engineering biomaterials with tailored properties and functionalities by modulating the pathways of supramolecular polymerization. We have also shown that the energy landscape of supramolecular polymerization can be manipulated *via* light-matter strong coupling, without any chemical or real photon as input. Intrigued by these recent observations, we employed light-matter strong coupling to control LLPS driven by non-covalent high aspect ratio supramolecular polymers. Studies using confocal microscopy, atomic force microscopy, and dynamic light scattering revealed that the energy landscape of the supramolecular polymerization of ureido-pyrimidinone glycine (UPy-Gly) fibrils is modified when the vibrational bands of the molecular components are strongly coupled to the optical mode of the Fabry–Perot cavity, leading to the deceleration of LLPS kinetics. Moreover, strong coupling persists in retarding LLPS kinetics even in the presence of a macromolecular crowder, however the effect is mitigated by the crowder. This offers insights into the fundamentals of strong coupling. Additionally, these results reinforce the finding that a critical fibril length is required for LLPS initiation. This study underscores the potential of light-matter strong coupling in tuning the behavior and assembly of supramolecular systems.

## Abstract 2

Liquid–liquid phase separation under vibrational strong coupling.

## Graphical abstract

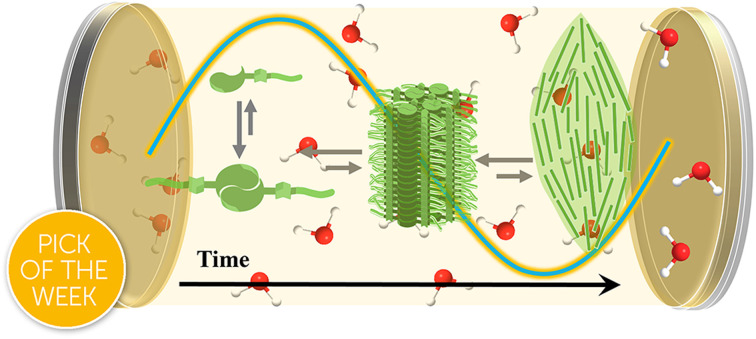

## Introduction

Liquid–liquid phase separation (LLPS) is a phenomenon that occurs when a homogeneous solution containing one or more components spontaneously separates into two distinct liquid phases.^1,2^ The resulting liquid condensates play a key role in a wide range of biological processes, including the modification of biochemical reaction rates, the formation of subcellular structures, the regulation of gene expression and macromolecular folding state.^3,4^ Perturbations in environmental factors like pH, concentration, temperature and ionic strength can influence the LLPS of biopolymers and lead to the formation of toxic aggregates implicated in diseases such as sickle-cell anemia, cancer and Parkinson's.^5–7^ Recently, we demonstrated that high aspect-ratio supramolecular polymers can also undergo LLPS, driven by the maximization of translational entropy and further accelerated by a macromolecular crowder.^8^ When the critical fibril length required for LLPS is achieved during supramolecular polymerization, the homogeneous solution undergoes phase separation to form a heterogeneous solution. The anisotropic alignment and rigidity of supramolecular fibrils leads to ellipsoidal structures, called tactoids. LLPS in supramolecular polymerization offers new possibilities for modulating the properties and functions of liquid condensates by controlling the assembly pathways of supramolecular polymers.^9^

**Fig. 1:**
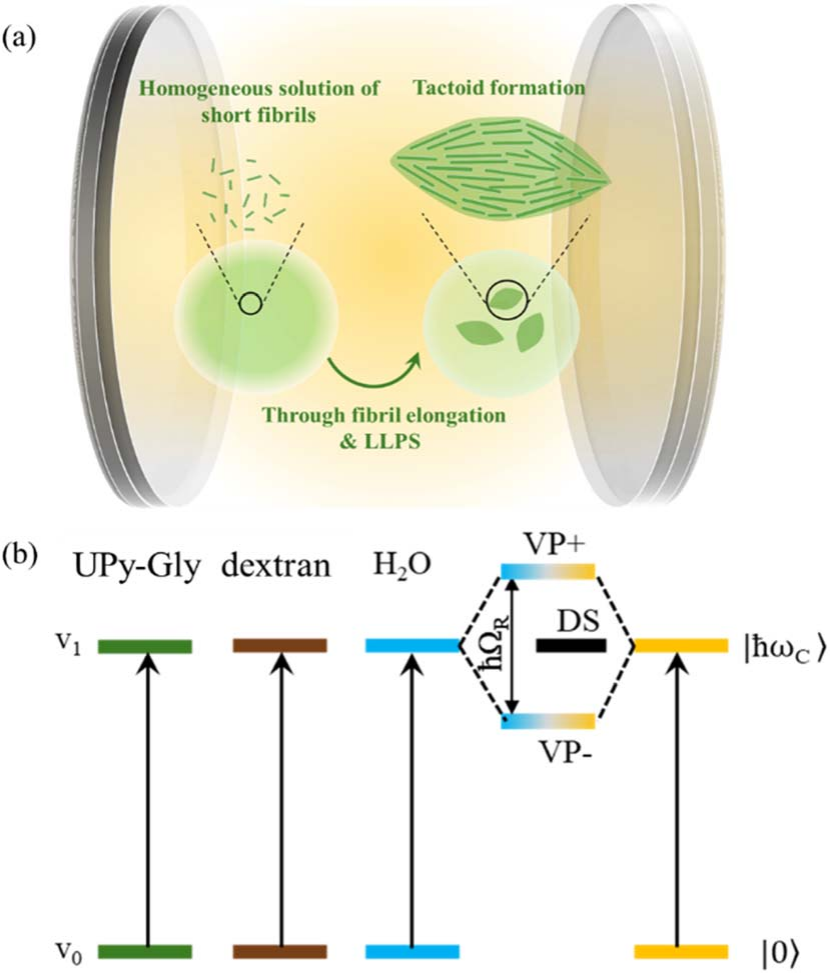
Schematic illustration of (a) LLPS driven by supramolecular polymerization studied in a Fabry–Perot (FP) cavity and (b) strong coupling condition, wherein the vibrational bands of the solutes (UPy and dextran) are coupled to the optical mode of the FP cavity through the cooperative effect mediated by the vibrational modes of the solvent (water). This leads to the formation of vibro-polaritonic states, including the bright states (VP+, VP−) and the dark states (DS). The upper and the lower polaritonic states (VP+ and VP−), are separated by an energy difference of Rabi splitting (ħ*Ω*_R_).

Supramolecular polymers have great potential for developing novel functional materials. Since supramolecular polymer systems are controlled by non-covalent and reversible interactions, it is possible to manipulate the supramolecular arrangement through various external factors and stimuli such as solvent composition, light, pH and temperature.^10–14^ We have recently demonstrated that a new paradigm, light-matter strong coupling, can also be employed to access different states of supramolecular polymerization, by simply tuning the optical mode in and out of resonance with molecular transitions, without relying on chemical or real photons as inputs.^15^ For over a decade, it has been shown that light-matter strong coupling modifies the interactions between solute, solute–solvent, and solvent molecules, thereby altering the chemical energy landscapes.^15–24^ For any system to be in the strong coupling regime, the exchange of virtual photons between matter and the resonant optical mode must be faster than the decay processes, leading to the formation of hybrid light-matter states, called polaritonic states. Due to the interaction with zero-point energy (or vacuum) fluctuations, such a coupling is possible even in the dark. Strong light-matter coupling can be achieved by coupling either electronic transitions (electronic strong coupling, ESC) or vibrational bands (vibrational strong coupling, VSC) to the optical mode. Under VSC, vibro-polaritonic states are formed, which includes the bright states and the dark states (DS). The bright states, the upper (VP+) and lower (VP−) polaritonic states, are separated by an energy difference known as Rabi splitting (ħ*Ω*_R_), as illustrated in Fig. 1.

**Fig. 2:**
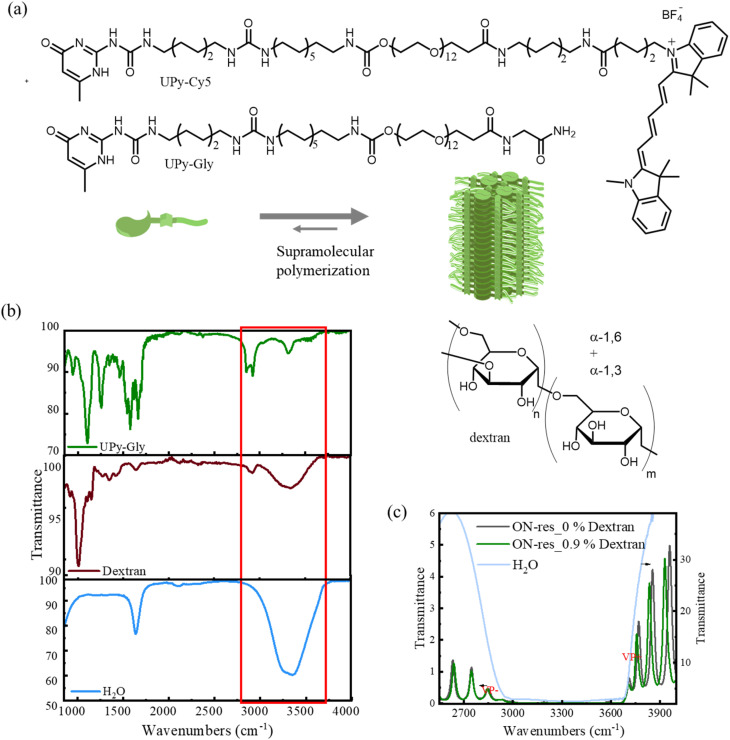
(a) Molecular structure of UPy-Gly, UPy-Cy5 and dextran, and the schematic illustration of UPy-Gly and the corresponding supramolecular polymer. (b) FT-IR transmission spectra of UPy-Gly, dextran and water. The highlighted peaks represent the overlapping vibrational bands between the solutes and the solvent that are necessary for cooperative coupling. (c) FT-IR spectra showing the ON-resonance condition of the 1% UPy-Gly aqueous solution with and without dextran.

Recent works have emphasized the impact of VSC on non-covalent interactions, and consequently, on various supramolecular systems.^15,17,20,21,23,25,26^ These studies inspired us to explore the effect of VSC on LLPS driven by supramolecular polymerizations, given that LLPS is sensitive to the perturbations in solute–solute, solute–solvent and solvent–solvent interactions.^8,27^ Here, we study the effect of VSC on the kinetics of LLPS through the study of the supramolecular polymerization in an optical cavity of ureido-pyrimidinone glycine (UPy-Gly, molecular structure is shown in Fig. 2a). The homoditopic UPy units form dimers, which are stabilized by strong self-complimentary quadruple hydrogen-bonding.^28,29^ Through π–π stacking and hydrogen bonding between the flanking urea groups, the UPy dimers assemble into long one-dimensional supramolecular polymers and rather rigid fibrils in time. In this study, we achieved strong coupling condition by coupling the O–H stretches of water, UPy units and the crowding agent to the optical mode. Our results, confirmed by confocal and atomic force microscopy (AFM) studies, show that VSC suppresses UPy fibril growth, leading to slower LLPS kinetics. Additionally, dynamic light scattering (DLS) was utilised to probe the growth of UPy fibrils under VSC, revealing its slow elongation and decelerated LLPS kinetics.

## Results and discussion

### Vibrational strong coupling of UPy-Gly *via* cooperative effect

Our recent report has demonstrated that a homogeneous solution of water-soluble UPy-Gly supramolecular polymers transitioned into a heterogeneous solution overnight, driven by fibril elongation.^8^ The same aqueous solution (pH = 7.6 ± 0.2, 0.25 × phosphate-buffered saline (PBS), supramolecular polymerization of 1 wt% of UPy-Gly) was studied in a Fabry–Perot (FP) cavity as illustrated in Fig. 1, and the results were then compared to control samples. Less than 0.1 mol% of the UPy-Cy5 dye (molecular structure is shown in Fig. 2a, whose synthesis is reported in the previous publication^8^) is used to track the UPy-Gly through co-assembly in the fibrils. The 1% UPy-Gly aqueous solution was injected into the Specac microfluidic cell (Fig. S1a). Gold-coated, IR transparent BaF_2_ substrates were insulated by a water-insoluble poly(methyl methacrylate) (PMMA) film and separated by a 25 μm Mylar spacer. These substrates were then assembled into the microfluidic cell and tuned to achieve strong coupling condition. For any system to be in the strong coupling regime, Rabi splitting (*Ω*_R_) must be larger than the full width half maximum (FWHM) of the optical mode and the molecular transition, while *Ω*_R_ depends on the concentration (*C*), *i.e.*
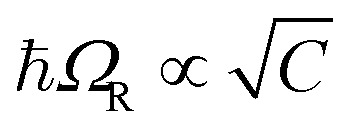
. Due to the limitations of solute concentration (1 wt% of UPy-Gly) in the solution, it is impossible to directly couple the solutes at the current concentration. Instead, the strong coupling condition, referred to hereafter as the ON-resonance cavity, is achieved *via* the cooperative effect. As illustrated in Fig. 1b, under cooperative coupling, when the vibrational bands of the solute and solvent overlap, the solute molecules can be coupled to the optical mode through coupling of the solvent molecules.^15–18,20,21,24,25^ As can be seen from Fig. 2b, UPy-Gly molecules have hydroxyl and urea groups with peaks around 3292 cm^−1^, aliphatic C–H stretches around 2850 cm^−1^ and 2920 cm^−1^ ^30^ and water has a very broad absorption band from 3000 cm^−1^ to 3600 cm^−1^. Under cooperative coupling, when the vibrational modes of the solvent (O–H stretches of water) are strongly coupled to the optical modes (21st–24th) of the FP cavity with the free spectral range (FSR) of 145.6 cm^−1^, the solute (UPy-Gly) is also strongly coupled, since its vibrational bands are at a similar frequency. Rabi splitting is ∼871 cm^−1^ (VP+ ∼3714 cm^−1^ and VP− ∼2843 cm^−1^). Fig. S2a shows that the solution is strongly coupled over the investigated time frame.

**Fig. 3:**
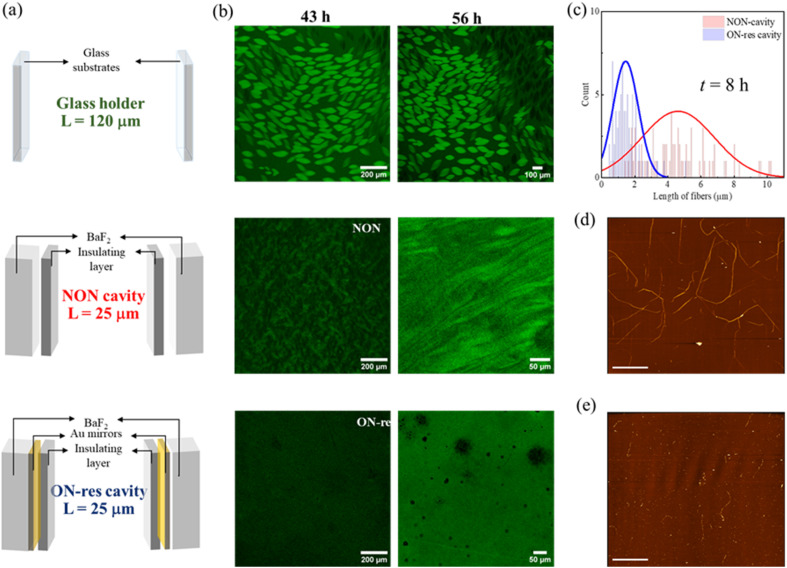
(a) Schematic illustration of glass holder, NON- and ON-resonance cavities. (b) CLSM images tracking the transition from homogeneous solution to liquid–liquid phase-separated solution followed by the growth of tactoids in the aqueous solution of 1% UPy-Gly supramolecular fibrils (labelled with 0.02 mol% of UPy-Cy5) in 0.25 × PBS, pH = 7.5 and 0% dextran in glass holder, NON- and ON-resonance cavities. (c) The corresponding fibril length distribution in the NON- (red) and ON-resonance (blue) cavities prior to phase separation was determined through the AFM images shown in (d) NON- and (e) ON-resonance cavities. AFM images, scale bars represent 10 μm.

Multiple cavity modes are coupled to the broad O–H stretching vibrations of water, and the system is in the ultra-strong coupling regime.^20^ Hence, the typical control experiment involving an OFF-resonance cavity, in which the optical mode is completely detuned from the vibrational bands, is impossible. Nevertheless, the following control experiments with NON- and single-mirror cavities address the potential artifacts caused by physical confinement and hydrophobic insulating film. The NON-cavity is fabricated by spin-coating the water-insoluble PMMA film directly onto BaF_2_ substrates. Different conditions (NON- and ON-resonance cavities) are also compared to the glass holder setup,^8^ wherein glass substrates are separated by a 120 μm imaging spacer (Fig. 3a).

### LLPS kinetics and supramolecular polymerization

The LLPS process was tracked using confocal laser scanning microscopy (CLSM). The CLSM results observed under different conditions – Glass holder, NON- and ON-resonance cavities (schematic illustrations in Fig. 3a) are studied and compared. As can be seen in Fig. 3b and S3a, the CLSM results of the aqueous solution with 1% of UPy-Gly show that tactoids are well-developed in glass holder at *t* = 24 h. While the solution is still homogeneous in NON-cavity, tactoids started to appear in NON-cavity only at *t* = 43 h and became more evident at *t* = 56 h but are distorted due to evaporation-induced flow. On the contrary, the solution remained mostly homogeneous in the ON-resonance cavity at *t* = 43 h and 56 h, indicating that LLPS is delayed under strong coupling conditions. Due to evaporation issues, we could not continue to track LLPS in the cavities after 56 h to determine when LLPS occurs under the strongly coupled condition. Note that due to the long working distance objectives required to image an intact cavity in the microfluidic cell (Fig. S1a), the cavities were removed from the cell for CLSM imaging and were later tuned back to the coupling condition. Nonetheless, since the system is in the ultra-strong coupling regime, all molecular components are strongly coupled to the optical mode. Additionally, the imaging time is typically much shorter than the incubation time.

To further understand the effect of VSC on supramolecular polymerization and LLPS, AFM images of samples prepared under various conditions were recorded and the fibril length distributions were analysed for those formed prior to the LLPS onset. Since LLPS typically occurs in the 1% of UPy-Gly aqueous solution at approximately *t* ∼10 h in the glass holder,^8^ AFM samples from NON-and ON-resonance cavities were prepared at around *t* ∼8 h (see Fig. 3d, e and S4a for the images). Distribution plots in Fig. 3c reveal significant differences between NON- (red) and ON-resonance (blue) cavities. The average length of UPy fibrils in the NON-cavity is 4.92 ± 3.31 μm, whereas in the ON-resonance cavity, it is 1.45 ± 0.76 μm. This indicates that VSC disfavours the growth of UPy supramolecular polymers *i.e.*, supramolecular polymerization occurs more slowly under the ON-resonance condition,^15^ thus elucidating the slower LLPS kinetics under the strongly coupled condition. Note that the fibrils may connect with each other, which could lead to an overestimation of their length.^8^

**Fig. 4:**
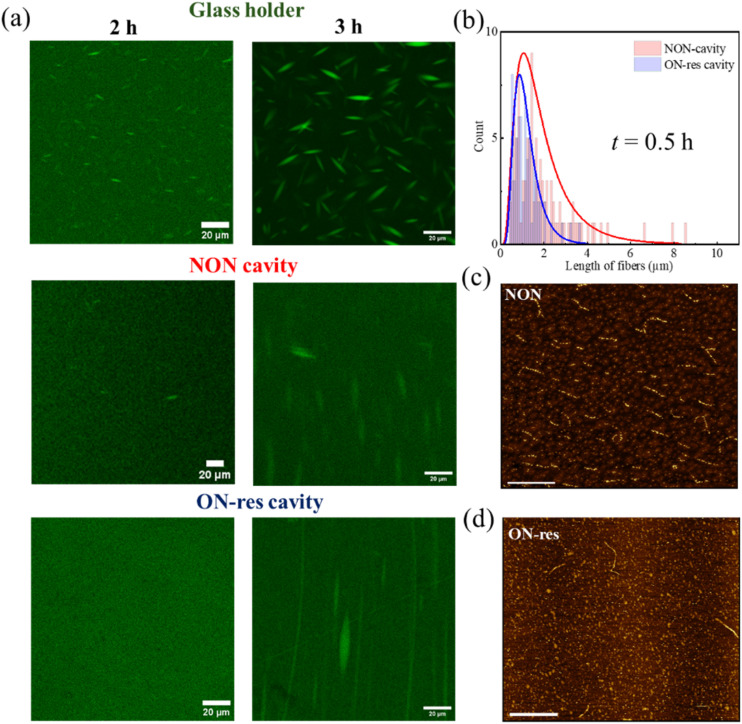
(a) CLSM images tracking the transition from homogeneous solution to liquid–liquid phase-separated solution followed by the growth of tactoids in the aqueous solution of 1% UPy-Gly, in 0.25 × PBS, pH = 7.5 and 0.9% dextran, in glass holder, NON- and ON-resonance cavities (b) the corresponding fibril length distribution in the NON- (red) and ON-resonance (blue) cavities prior to phase separation, tracked through the AFM images shown in (c) NON- and (d) ON-resonance cavities. AFM images, scale bars represent 10 μm.

To gain better insight into the decelerating effect of VSC, a macromolecular crowder, dextran (molecular structure shown in Fig. 2a), was introduced into the UPy-Gly aqueous solution. For achieving VSC, O–H stretch of dextran is strongly coupled to the 21st–26th optical modes of the FP cavity (FSR = 139 cm^−1^; Fig. 2c), in addition to the hydroxyl and urea stretches of other molecular components, *via* the cooperative coupling. The corresponding Rabi splitting is ∼864 cm^−1^ (VP− ∼2843 cm^−1^ and VP+ ∼3707 cm^−1^), and Fig. S2b and S6c show that the aqueous solutions with dextran (0.9% and 0.75%) is strongly coupled over the duration of our study. For the aqueous solution with 0.9% dextran, CLSM images reveal that LLPS of the solutions under strongly coupled condition are all slower than those in the glass holder and NON-cavity, as is clearly seen in Fig. 4a and S3b. However, the deceleration effect is less pronounced. The corresponding fibril length distribution (Fig. 4b), traced through AFM for the samples prepared at around *t* ∼ 0.5 h, reveals the average lengths in NON- (red) and ON-resonance (blue) cavity to be 0.98 ± 0.46 μm and 0.64 ± 0.27 μm, respectively (see Fig. 4c, d and S4b for the images). Additional control experiments performed in single-mirror cavities further confirm our observations (Fig. S5). Furthermore, a similar trend of retarded LLPS kinetics was observed for the aqueous solution with 1% UPy-Gly and 0.75% dextran under VSC, as can be seen in Fig. S6. The crowding effect influences intermolecular interactions, hydrophobic forces, hydrogen bonding and more generally van der Waals forces, and it also accelerates LLPS and supramolecular polymerization through the volume exclusion effect.^1,8,31^ In cavity quantum electrodynamics, it is known that light-matter strong coupling modifies the non-covalent interactions such as London dispersion forces and hydrogen bonding, thereby altering chemical and supramolecular energy landscapes.^15–18,23,32,33^ Therefore, ON-resonance condition might be disfavoring the solute–solute, solute–solvent and solvent–solvent interactions that are responsible for the elongation of UPy-Gly fibrils, even in the presence of the macromolecular crowder, though this effect is attenuated by the accelerating effect induced by the crowder.

Since all the cavity substrates are insulated by a hydrophobic PMMA layer, control experiments were carried out to compare the influence of substrate hydrophobicity on nucleation at the interface and hence, on LLPS kinetics. As shown in Fig. S7, LLPS kinetics was compared between bare glass holders which have exposed O–H stretches and those spin-coated with PMMA. For the 0% crowder solution, the LLPS kinetics is slowed down due to the PMMA–water interaction, which modified nucleation at the surface interface. In contrast, for the 0.9% crowder solution, the crowding effect nullifies the hydrophobic effect of the PMMA film, therefore, its effect on LLPS kinetics is negligible. Moreover, Fig. 3b, 4a and S3 indicate that the effect of physical confinement is not negligible. However, since the polymer-coated substrates and the path length are consistent across the NON-, ON-resonance and single-mirror cavities. Therefore, VSC is primarily responsible for the observed deceleration of the LLPS kinetics.

### Tracking the diffusion dynamics by DLS

In addition to the CLSM and AFM imaging, we performed *in situ* DLS measurements using a home-built setup (schematic illustration is shown in Fig. S8a), as VSC has been shown to modify the length of supramolecular polymers, and DLS is a sensitive probe of their concentration dynamics and the size of diffusing particles in the reacting medium. Therefore, DLS measurements were performed in NON- and ON-resonance cavities at two probing wavelengths ∼2π/*q* with the scattering wavevector *q* = (4π*n*/*λ*)sin(*θ*/2), where *n* is the refractive index and *λ* (=632 nm) the laser wavelength. To our knowledge, very few such experiments have been performed in microfluidic configuration.^34^ The relaxation function *C*(*q*, *t*) is usually obtained from the experimental intensity autocorrelation function *G*(*q*, *t*) recorded under homodyne conditions, where *C*(*q*, *t*) = [*G*(*q*, *t*) − 1]^½^.^8^ However, the scattered light from a 25 μm path length is strongly mixed with elastically scattered light leading to pure heterodyne conditions evidenced from the low amplitude of *C*(*q*, *t*) = [*G*(*q*, *t*) − 1].^35^ Deliberately, we chose low *q* and long laser wavelength *λ* (=632 nm) to shift the diffusive relaxation rates^8^ in the desirable intermediate time range of *C*(*q*, *t*), which reduces the noise and diminishes heating due to plasmonic absorption. Normal incidence of the incident laser light also excludes contribution from evanescent DLS(**x**). Note that a 25 μm spacer was intentionally selected for the cavity studies given that evanescent DLS contributes to the scattering curves at shorter path lengths, the associated low signal-to-noise ratio, and the strong influence of physical confinement under these conditions.

**Fig. 5:**
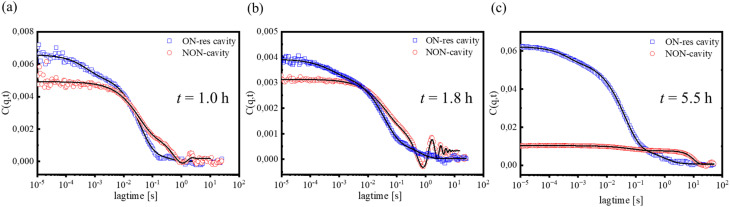
Comparison of the experimental relaxation function *C*(*q*, *t*) of 1% UPy-Gly, 0% dextran, 0.25 × PBS, pH = 7.5 solution in NON- (red circles) and ON-resonance (blue squares) cavities recorded at (a) *t* = 1.0 h, (b) *t* = 1.8 h, and (c) *t* = 5.5 h after annealing at 295 K. The main process of *C*(*q*, *t*) is represented (solid lines) with a stretched exponential, which is very similar for both cavities. An additional fast and a slower process distinguish the NON- and ON-resonance cavities, respectively (see text).

Fig. 5a–c displays experimental *C*(*q*, *t*) at a low *q* (=0.0038 nm^−1^) of the 1% UPy-Gly and 0% dextran solution in NON (red) and ON-resonance (blue) cavities at three different “polymerization” times [1.0 h (a), 1.8 h (b) and 5.5 h (c)]. The pattern of the relaxation function *C*(*q*, *t*) is distinct in the two cases with a common feature of the main intermediate decay represented by an almost exponential (*β* = 0.85) with a relaxation time, *τ* ≈ 32 ms (NON-cavity) or 36 ms (ON-resonance cavity). Based on the diffusive nature of *τ*^−1^ (=*Dq*^2^) (Fig. S8b and c), this process is assigned to the fibril translation diffusion (*D* ∼ 5 × 10^−12^ m^2^ s^−1^) in dilute solution,^8^ indicating similar fibril length distributions in both cavities at this time point. However, as time elapsed, the slow mode in NON-cavity shows a larger characteristic time (*τ* ≈ 40 ms) at *t* = 1.8 h due to fibril elongation through supramolecular polymerizations, while this mode for the ON-resonance cavity shows no obvious change compared to the earlier time. This trend is also observed at longer annealing period (at *t* = 5.5 h in Fig. 5c) as indicated by the slower *τ* ≈ 75 ms in the NON-cavity compared to *τ* ≈ 50 ms under ON-resonance. These results suggest that fibrils grow at a slower speed in ON-resonance cavity than in the NON-cavity, which is consistent with the AFM results (Fig. 4a). Note that the translation diffusion *D* of the fibrils depends on fibril diameter and persistence length, in addition to their length (8), in contrast to spherical particles.^36^

For the NON-cavity, *C*(*q*, *t*) displays an additional slow process (*τ* ≈ 10 s) with either oscillation (Fig. 5a and b) or contracted exponential (*β* = 2) shape (Fig. 5c) suggesting propagation concentration wave or deterministic motion, respectively. Notably, this kind of dynamics can only be observed under heterodyning conditions^36^ and can suggest heterogeneous polymerization in the confined space (25 μm) between two PMMA-coated BaF_2_ substrates. This peculiar slow process is not discernible for the ON-resonance cavity. Instead, as seen in Fig. 5, a weak and fast exponential relaxation with *τ*_f_ ≈ 0.5 ms at *t* = 1.0 h, 1.8 h and 5.5 h is observed. Both findings may be related to the effect introduced by light-matter strong coupling. The fast characteristic diffusion time is indicative of diffusive nature with a hydrodynamic size of *R*_H_ ∼1.2 nm. This might indicate the presence of sub-nanometer-sized water clusters or UPy oligomers detached from UPy fibrils, indirect evidence that VSC disfavors the formation of UPy fibrils. On the other hand, the coherent and collective strong coupling between the molecular vibrational transitions and the FP cavity modes could potentially homogenize the system,^22^ and account for the disappearance of the slow process in the ON-resonance cavity. This unexpected behavior of the present interacting system in the microfluidic optical cavity will be the subject of a separate study.

**Fig. 6:**
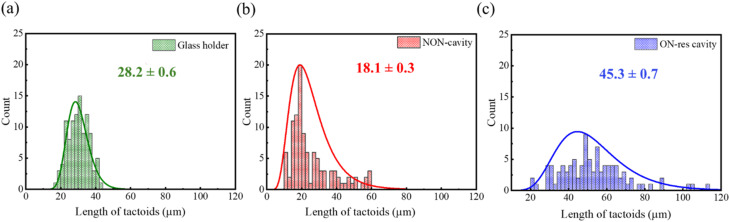
Comparison of the length of tactoids formed in the aqueous solution with 1% UPy-Gly, 0.9% dextran, 0.25 × PBS, pH = 7.5 in (a) glass holder, (b) NON- and (c) ON-resonance cavities.

Interestingly, we observed that tactoids grew larger in the ON-resonance cavity than in the glass holder or NON-cavity for the solution with 1% UPy-Gly and 0.9% dextran once the critical fibril length is reached (Fig. 6). However, we observed no change in the aspect ratio (Fig. S9). Both the NON-and single-mirror cavities consider the possible artifacts resulting from microfluidic evaporation and the directional flow in the Specac cell. Unfortunately, such a comparison is not possible for the 0% dextran solution due to time constraints and evaporation issues. The presence of larger tactoids further indicates that nucleation proceeds more slowly.

## Conclusion

The effect of VSC on LLPS driven by the elongation of non-covalent high-aspect ratio supramolecular polymers is studied. The CLSM results indicate that LLPS kinetics is decelerated when the O–H stretch of water, UPy-Gly and dextran are strongly coupled to the optical modes of the FP cavity, with the coupling of UPy-Gly and dextran enabled through cooperative effect. Moreover, AFM and DLS results demonstrate that the deceleration of LLPS is due to the slowed fibril elongation under VSC. The introduction of the macromolecular crowder alleviates the decelerating effect on LLPS kinetics, indicating the diametric effects of the crowding agent and the strong light-matter coupling at play. Additional control experiments reveal the potential effects of surface hydrophobicity and physical confinement. These results indicate that VSC affects the intermolecular and intramolecular interactions, altering the energy landscape of UPy-Gly supramolecular polymerization, and thereby modifying the LLPS kinetics. As previously reported, VSC modifies the thermodynamic parameters and the chemical energy landscape.^37^

## Materials and methods

### Fabrication of glass holder, ON-resonance, NON- and single-mirror cavities

The control setup, glass holder was prepared by assembling two cover glasses separated by a 120 μm Grace Bio-Labs SecureSeal imaging spacer (1 well, diameter × thickness: 9 mm × 0.12 mm).

BaF_2_ (IR transparent) windows, tunable microfluidic cells, and 25 μm Mylar spacers were purchased from Specac. The windows were sputtered with 10 nm of Au in a Quorum Q150T Plus turbomolecular coater and then spin-coated with a 4% (w/w) PMMA in toluene at 3000 rpm. The Au mirrors were separated by a 25 μm Mylar spacer and assembled into the microfluidic cell. Since the O–H stretch of water is broad, the system is always in strong coupling condition.

The optical cavities were tuned such that the sweet spot was approximately 85–90% of the total area, so that the CLSM imaging would be possible over most of the surface.I
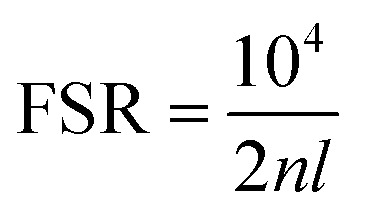
Based on the refractive index (*n*) of the solvent and the spacing between the mirrors (*l*), the free spectral range (FSR) can be calculated using eqn (I).

For NON-cavity experiments, the substrates were prepared by spin-coating PMMA directly onto BaF_2_ windows. The additional reference, single-mirror cavity was fabricated by replacing one of the substrates in ON-resonance cavity by a bare BaF_2_ window coated with PMMA. NON- and single-mirror cavities take care of the artifacts contributed by the insulation film, physical confinement, or Au mirrors.

### Sample preparation

UPy-Gly solution without dextran: a freshly prepared stock solution of UPy-Gly was used every time. To prepare a 4 wt% of stock solution, UPy-Gly solid was dissolved in the PBS buffer and a calculated amount of 1 M NaOH, to make the final pH higher than 11. The solution was then heated and stirred at 75 °C for 15 min. Subsequently, a calculated amount of 600 μM UPy-Cy5 in methanol was added to the stock solution, bringing the final concentration to 7.2 μM (UPy-Cy5). Finally, 1 M HCl was added to adjust the pH to the target value.

UPy-Gly solution with dextran: the freshly made UPy-Gly stock solution was immediately added to the prediluted PBS solution and mixed. Then, a 10 wt% of dextran solution with 0.08 mol% of dextran-FITC was added to reach to the required concentration. The mixture was then vortexed for 20–30 s.

### Confocal imaging

Due to the need of long working distance objectives to image the intact cavity in the Specac microfluidic cell, the cavity was removed, imaged and then placed back into the microfluidic cell. Fluorescent images and videos were acquired using a Leica TCS SP8 microscope in the confocal mode with 10× and 20× at resolutions of 512 × 512, 1024 × 1024 and 2048 × 2048 pixels. A 638 nm (Cy5) laser was used for imaging.

### Preparation of samples for AFM imaging

After opening the cavity, 1 μl of the solution was extracted from the top of the substrates and diluted a thousandfold in distilled water. To prepare dry samples for tapping mode imaging, 5 μl from the diluted solution was spin-coated onto a freshly cleaved mica substrate of size 1.0 × 1.0 cm^2^ at a speed of 2000 rpm for 1 min.

### DLS in-built setup

The microfluidic cell, with the cavity intact, was placed directly into the sample holder for *in situ* DLS measurements in a home-built setup. This setup consisted of a of compact goniometer system with a double detector, light-scattering electronics, tau digital correlator (ALV7004, Langen, Germany) and a 632 nm wavelength laser (iFLEX Viper, Pointsource, UK), as shown in Fig. S8a. DLS data were collected at two *q*'s.

## Author contributions

E. W. M. conceived the project. K. J. and H. F. designed the project and conducted the cavity and LLPS studies. J. v. d. T. performed the AFM measurements, and F. R. conducted control experiments. W. S. and G. F. performed and analyzed the DLS measurements. All authors contributed to the manuscript and approved the final version of the manuscript.

## Conflicts of interest

There are no conflicts to declare.

## Supplementary Material

SC-OLF-D5SC04149J-s001

## Data Availability

All data are given in the manuscript and the SI. The supplementary information provides additional experimental results that support the main finding. See DOI: https://doi.org/10.1039/d5sc04149j.
