# Supplementary material for: Impact of vibrational strong coupling on liquid–liquid phase separation in supramolecular polymers
Source: Chem Sci. 2025 Aug 7;16(37):17139–47. doi: 10.1039/d5sc04149j (PMC12394911; doi:10.1039/d5sc04149j)
Supplement: SC-016-D5SC04149J-s001 [file SC-016-D5SC04149J-s001.pdf]

Supplementary Information (SI) for Chemical Science.
This journal is © The Royal Society of Chemistry 2025

Supporting Information for

Impact of Vibrational Strong Coupling on Liquid-Liquid Phase Separation in Supramolecular Polymers

Kripa Joseph, [1†]*Hailin Fu,*[2†] *Joost J. B. van der Tol,* [1] *Werner Steffen,* [3] *Feixia Ruan,* [2,4] *George Fytas,*[*] [3,5,6] *and E. W. Meijer* [*1,3]

[1]Institute for Complex Molecular Systems, Laboratory of Macromolecular and Organic Chemistry, Eindhoven University of Technology, PO Box 513, 5600 MB, Eindhoven, The Netherlands.

[2]Department of Materials Science and Engineering, Department of Chemistry, Research Center for Industries of the Future, Westlake University, 310030, Hangzhou, China

[3]Max Planck Institute for Polymer Research, Mainz, Germany.

[4]School of Material Science and Engineering, Zhejiang University, 310030, Hangzhou, China.

[5]Faculty of Physics, Adam Mickiewicz University, Uniwersytetu Poznanskiego 2, 61-614 Poznan, Poland.

[6]Institute of Electronic Structure and Laser, FO.R.T.H, Heraklion, Greece.

TABLE OF CONTENTS

1. Microfluidic cell and illustration of a single mirror cavity S2
2. Time-dependent FT-IR spectra S2
3. Control experiments: Effect of physical confinement on LLPS kinetics S3
4. AFM images S4
5. Control experiments using single mirror cavity experiments S4
6. Effect of VSC on LLPS for UPy-Gly1% dextran 0.75% S5
7. Effect of PMMA on LLPS kinetics S6
8. DLS measurements S7
9. Aspect ratio of tactoids in optical cavities S7

1. Microfluidic cell and illustration of single mirror cavity

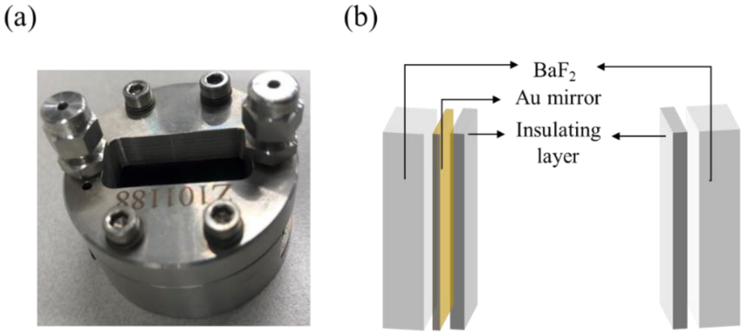

Figure S1: (a) Image of a tunable Specac microfluidic cell. (b) Schematic illustration of a single-mirror cavity.

2. Time-dependent FT-IR spectra of ON-resonance cavity.

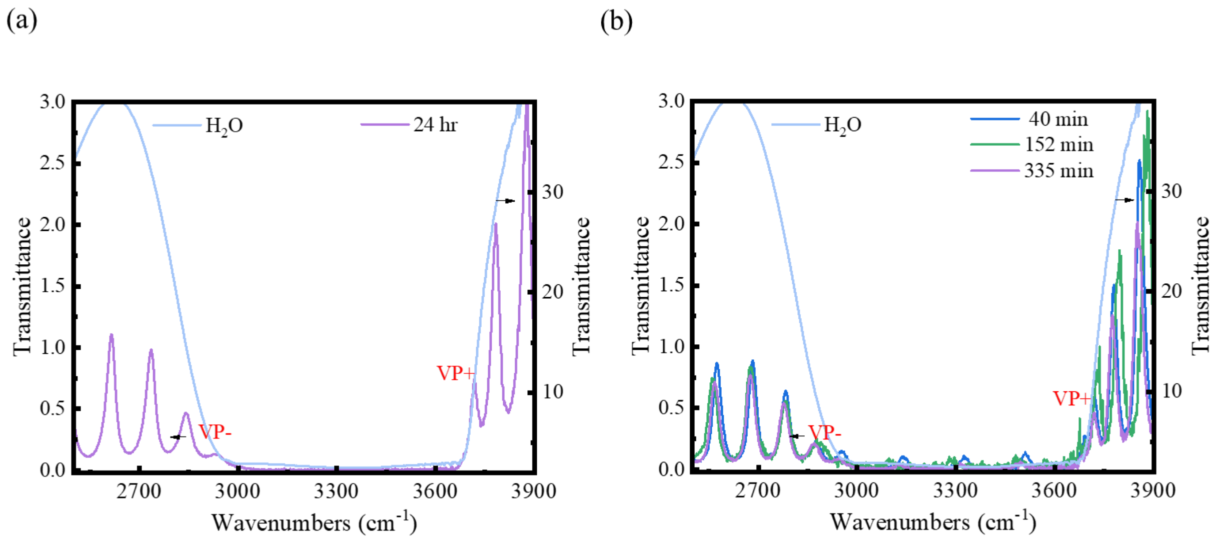

Figure S2: Time-dependent FT-IR spectra of ON-resonance cavity with the solution of 1% UPy-Gly, 0.25xPBS, pH = 7.5 with (a) 0% dextran and (b) 0.9% dextran.

3. Control experiments: Effect of physical confinement on LLPS kinetics

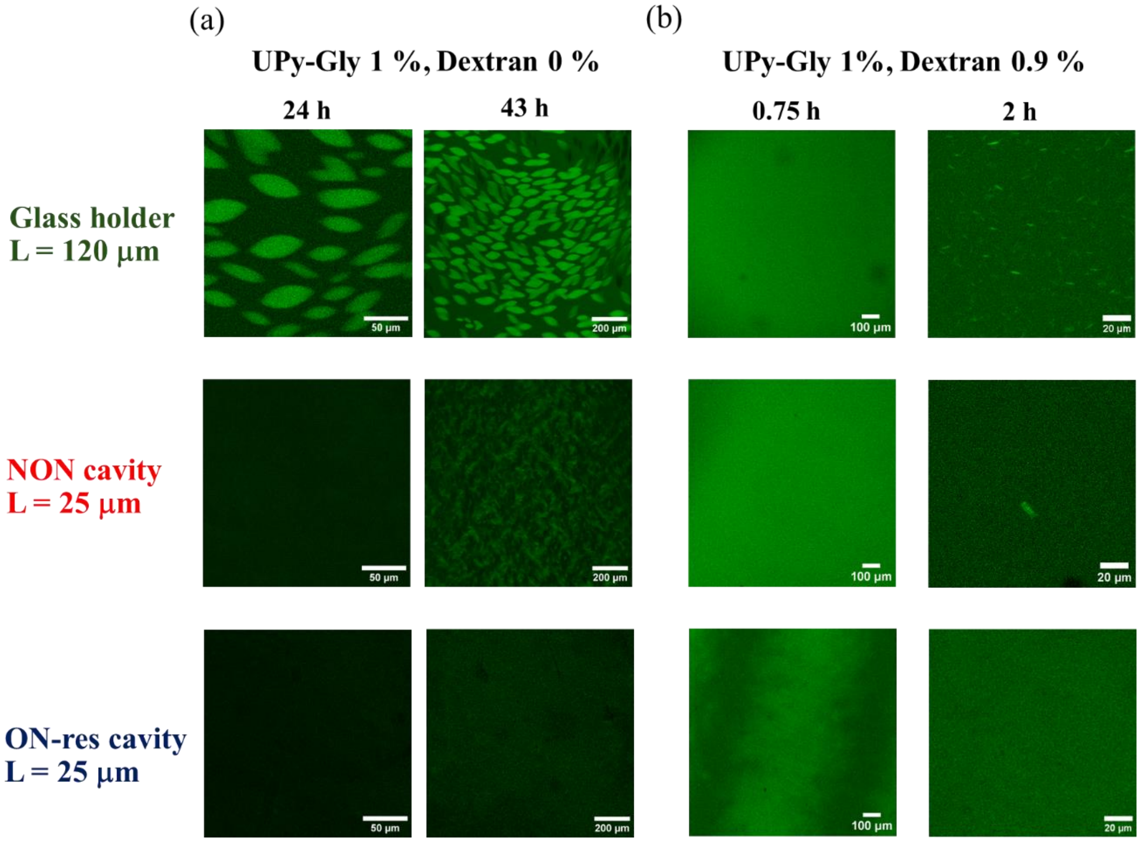

Figure S3: **Effect of physical confinement on LLPS kinetics.** CLSM images tracking the transition from homogeneous solution to liquid–liquid phase-separated solution followed by the growth of tactoids over the 1 wt% UPy-Gly (supramolecular polymers were labelled with 0.02 mol% of UPy-Cy5, PBS × 0.25, pH = 7.5 (a) 0% dextran (b) 0.9% dextran.

At *t* = 24h, the aqueous solution is still homogeneous in NON-cavity and ON-resonance cavity, while the tactoids are well-developed in the glass holder.

4. AFM images

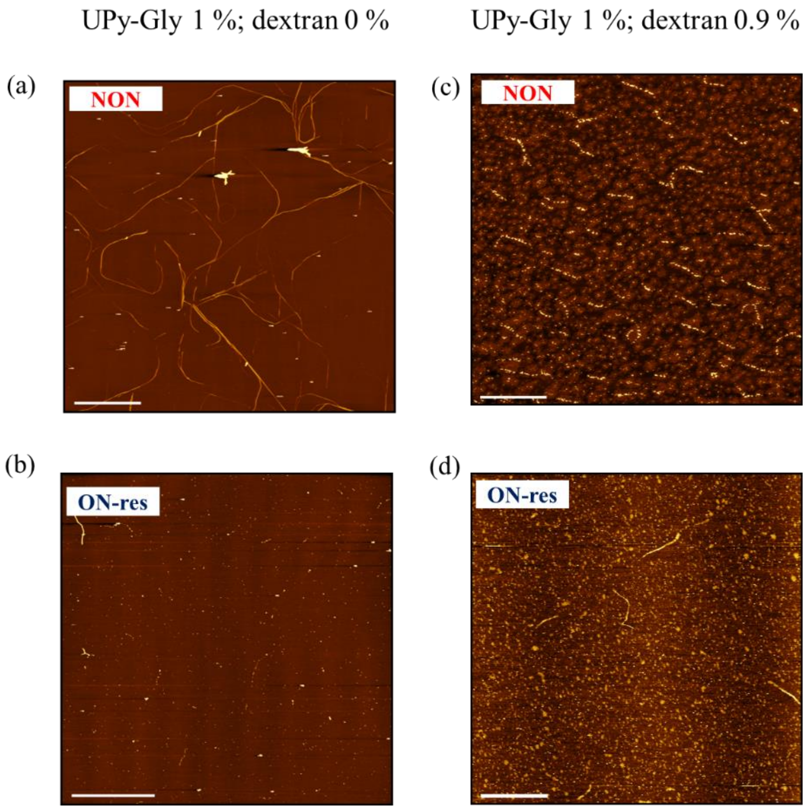

Figure S4: Representative AFM images for fibril length distribution plots in 1% UPy-Gly, PBS x 0.25, pH = 7.5, 0% dextran (a) NON- and (b) ON-resonance cavities; 1% UPy-Gly, PBS x 0.25, pH = 7.5, 0.9% dextran (c) NON- and (d) ON-resonance cavities. Scale bars represent 10 μm.

5. Control experiments using single-mirror cavity experiments

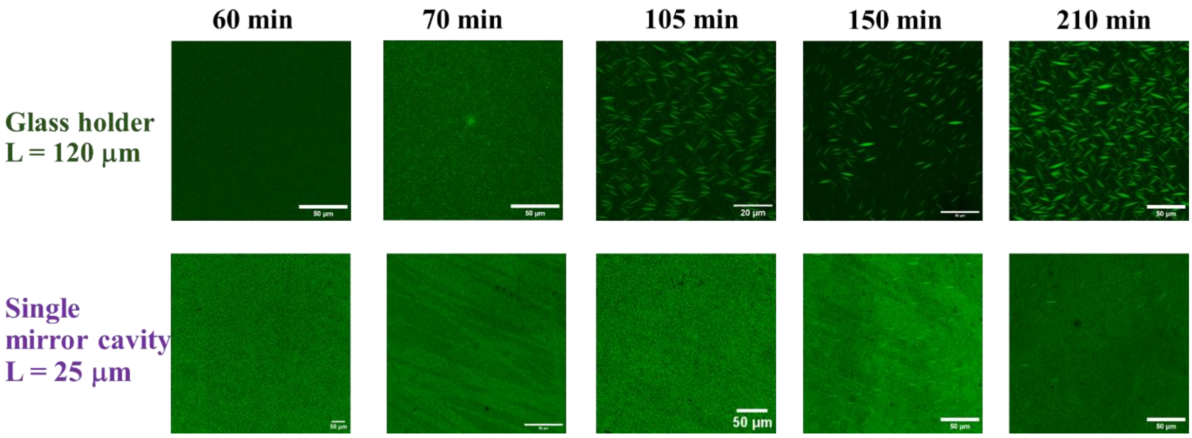

Figure S5. Single-mirror cavity experiment: CLSM images of tactoid formation in Control (glass holder) and single-mirror cavity studies done with the aqueous solution of 1% UPy and 0.9% dextran. Similar to NON-cavity experiments, tactoids start to appear after 2 h in single-mirror cavity setup.

6. Effect of VSC on LLPS for UPy-Gly1% dextran 0.75%

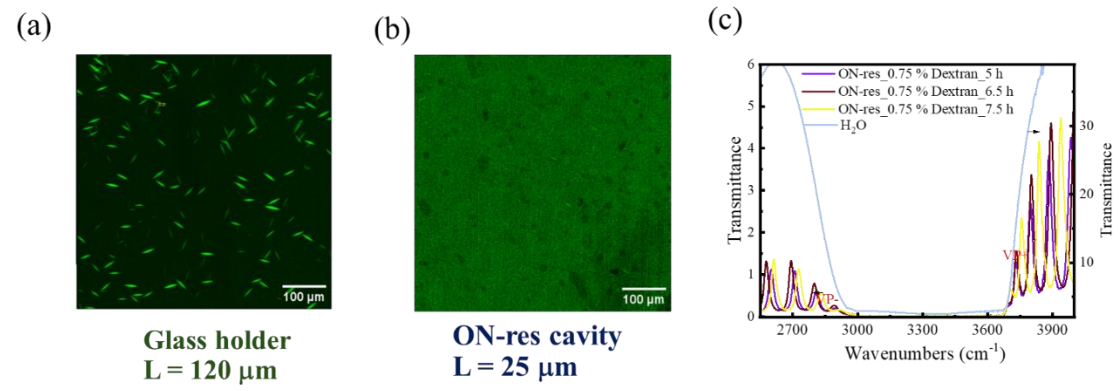

Figure S6: CLSM images tracking the transition from homogeneous solution to liquid–liquid phase-separated solution followed by the growth of tactoids over the UPy-Gly (1 wt%), supramolecular polymers were labelled with 0.02 mol% of UPy-Cy5, PBS × 0.25, pH = 7.5, 0.75% dextran, t = 7.5 h. Note that the aqueous solution is still homogenous in the ON-resonance cavity at t = 7.5 h.

7. Effect of PMMA on LLPS kinetics

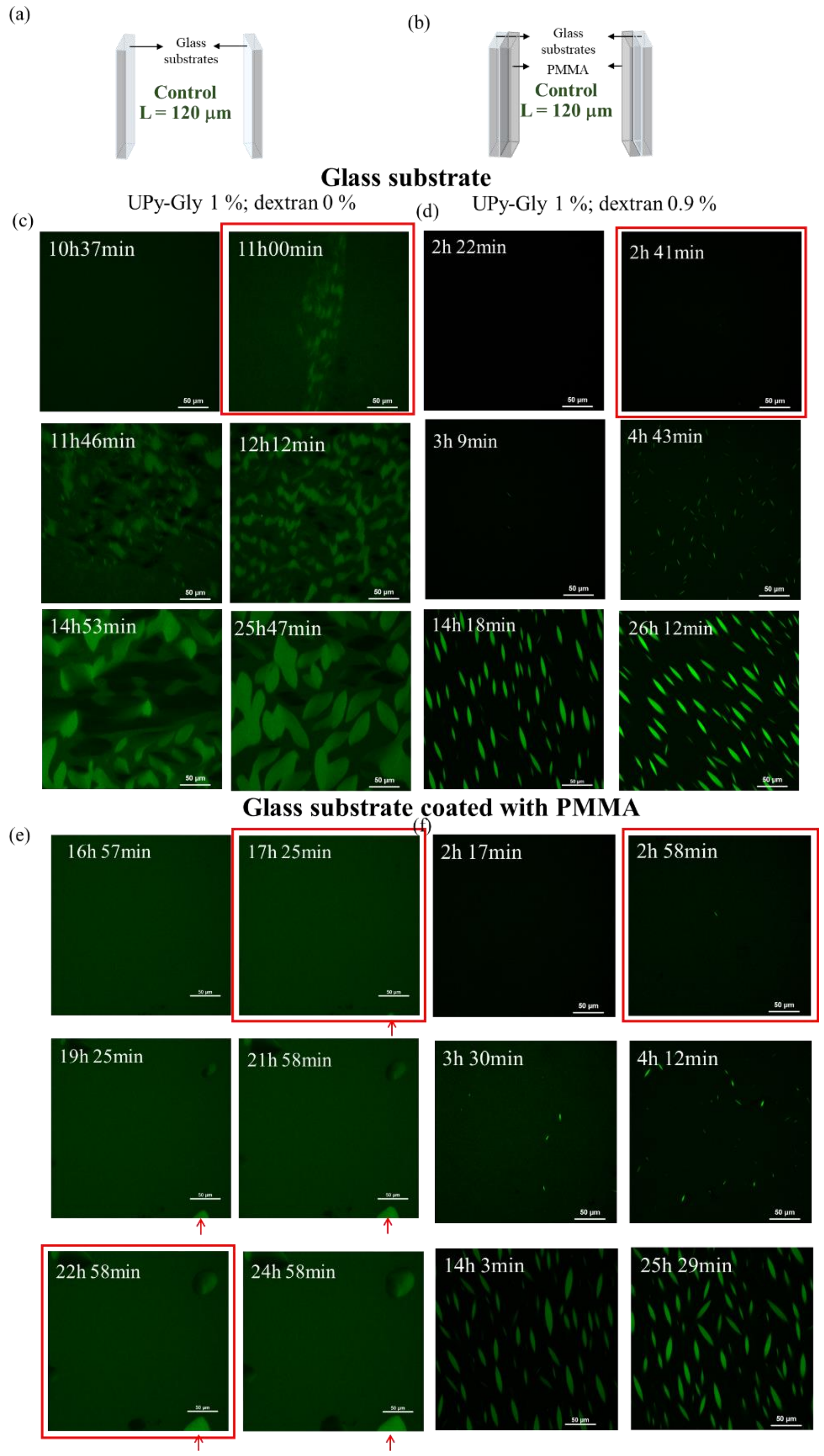

Figure S7: Effect of hydrophobicity of protection layer (PMMA) on nucleation at the interface: Schematic illustration of (a) bare glass substrates and (b) the substrates coated with PMMA. CLSM images comparing the phase separation in bare glass substrates with (c) 0% and (d) 0.9% dextran. CLSM images comparing the phase separation in PMMA-coated glass substrates with (e) 0% and (f) 0.9% dextran. Note that in the presence of 0.9% dextran, the effect of hydrophobicity of PMMA on LLPS kinetics is negligible.

8. DLS measurements

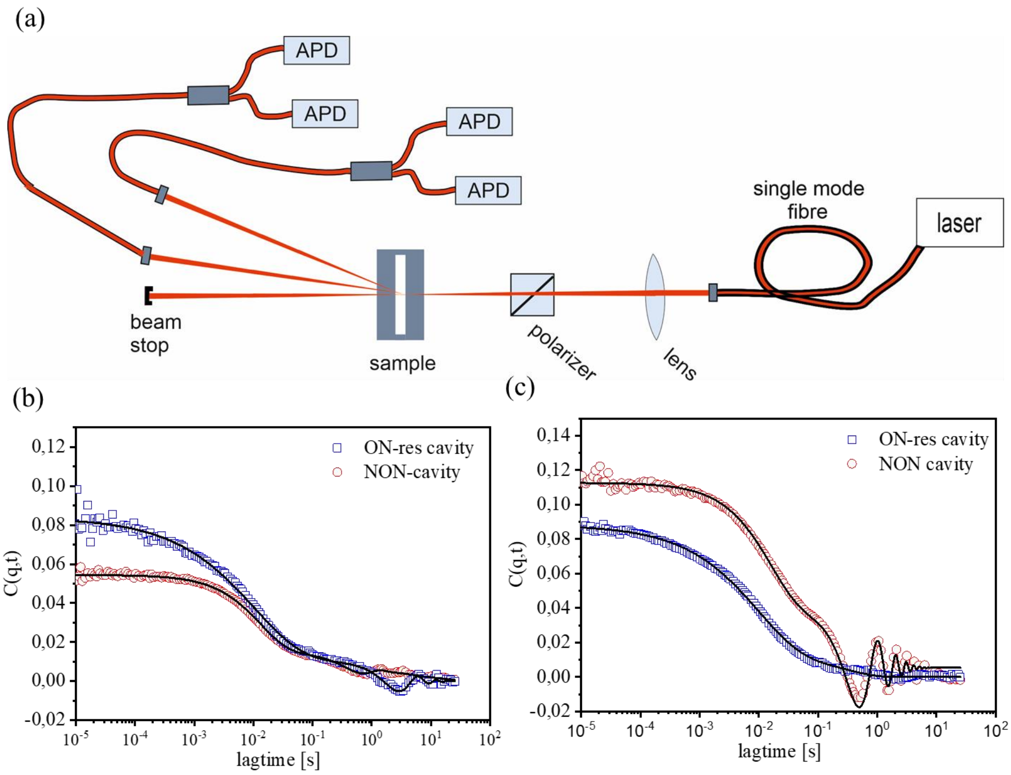

Figure S8: (a) Dynamic light scattering setup. Relaxation function C(q,t) for the concentration fluctuations at higher q than that in Figure 5. NON- and ON-resonance cavities at (b) t = 1 h and (c) t = 1.8 h polymerization time. The main process is represented by C(q,t) = aexp[/(t/τ)$^{\beta}$.

For NON-cavity, τ =18 ms, β=0.77 at both t = 1 h and t = 1.8 h (Figure S8b and c), whereas the oscillation at long times at 1.8 h conforms to cos (ω_ot) exp(-t/τ_o) with the propagation ω_o = 6.1s^{-1} and the damping τ_o = 2.2 s. Note that at the lower q of Figure 5, ω_o = 3.8 s^{-1} is accordingly slower corresponding to a speed of ~ 2 μm/s The damping τ_o (= 2,2 s) is q-independent.

The main process in both ON- and NON- cavity is diffusive as its rate 1/τ(q)~q^2 . The two fixed values of q were estimated using either the fiber translation diffusion, D = 5 μm^2/s or the diffusion time in a dilute latex sphere suspension.

9. Aspect ratio of tactoids in optical cavities

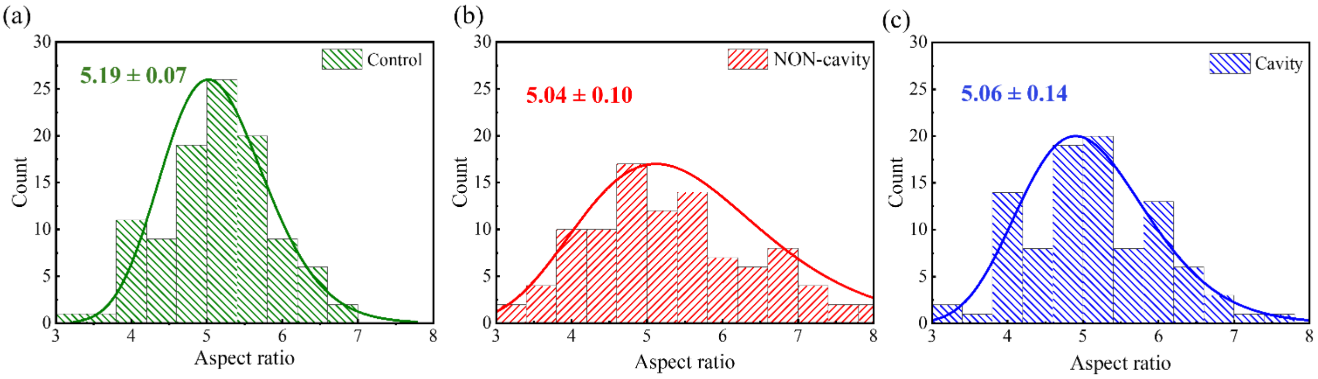

Figure S9. Comparison of aspect ratio (ratio of long axis to short axis) of tactoids formed in Glass holder, NON- and ON-resonance cavities.
